# Prevalence of nonsuicidal self-injury in chinese middle school and high school students: A meta-analysis

**DOI:** 10.1097/MD.0000000000012916

**Published:** 2018-10-19

**Authors:** Junjie Lang, Yingshui Yao

**Affiliations:** School of Public Health, Wannan Medical College, Wuhu, Anhui, China.

**Keywords:** meta-analysis, NSSI, self-injury, students

## Abstract

Recent studies have revealed a high rate of nonsuicidal self-injury (NSSI) behavior in Chinese students, but quantitative syntheses of pooled prevalence are sparse. There have been several NSSI prevalence meta-analyses in other populations. However, given the existence of cultural diversity, racial difference, educational system difference and so on, these results may not be ideal for Chinese populations. Furthermore, the above-mentioned meta-analyses did not include Chinese database which may have led to unintentional bias. Thus, we conducted this meta-analysis to estimate the prevalence of NSSI in Chinese middle-school students.

The databases searched included PubMed, Embase, Web of Science, CBM (Chinese database), Chinese National Knowledge Infrastructure (CNKI), Wanfang Data (Chinese database) and the Weipu database (Chinese database). The search terms included: self-injury/self-harm/self-abuse/nonsuicidal self-injury/deliberate self-harm, adolescen∗/youth/teen/students, and China/Chinese. All relevant articles published between January 2000 to November 2017, in either Chinese or English, were included. Two investigators were engaged in this process, and any disagreements were settled by a third investigator. A random effects model was then used to calculate the pooled prevalence.

A total of 420 studies with 160,348 participants were retrieved. The pooled prevalence was 22.37% (95% CI: 18.84%–25.70%). Substantial heterogeneity in prevalence estimates was revealed. Subgroup analyses showed that the pooled estimate of prevalence of life time NSSI was 14.5% (95%CI: 0.06%–22.7%), and 6–24 months NSSI was23.3% (95%CI: 20.5%–26.1%). The prevalence for males was 20.6% (95% CI: 16.1%–25.0%), and for females was 21.9% (95% CI: 17.6%–26.2%).

The prevalence of NSSI in Chinese middle-school students is relatively high. More attention should be paid to the current situation.

## 1 Introduction

Nonsuicidal self-injury (NSSI), which is defined as direct, deliberate damage to one's own body tissue without suicidal intent, is an alarmingly prevalent and dangerous phenomenon.^[1]^ A recent meta-analysis showed that prevalence rates of these behaviors range from 15.9%–20.5% among adolescents compared with 2.5%–5.4% among adults.^[2]^ Some articles have also suggested that NSSI is increasing in young people.^[3,4]^ In addition to being dangerous in its own right, NSSI may be a risk factor for future suicidal behaviors.^[5–8]^ The risk of suicide in the first year after self-injury was 66 times the annual risk of in general population, the risk of suicide after 5,10 and 15 years from self-injury is 1.7%, 2.4% and3.0%, respectively.^[9]^

In recent years, self-injury in physically and mentally immature adolescents has been the subject of intense public concern in China. Many investigations have been conducted into the prevalence of NSSI in Chinese middle-school students. However, owing to variations in sample size, design of study, outcome, and geographical area, the reported prevalence has varied greatly from a minimum of 6.4% to a maximum of 47.5%, even among homogeneous groups. Given this variation, it is difficult to fully understand the current situation with respect to NSSI.

There have been several NSSI prevalence meta-analyses in other populations.^[2,10]^ However, it is inapposite to apply the results from the above-mentioned meta-analyses to China, given the existence of cultural diversity, racial difference, educational system difference and so on.^[11]^ Furthermore, the above-mentioned meta-analyses did not include Chinese database which may have led to unintentional bias.

Therefore, we undertook a meta-analysis to estimate the prevalence of NSSI in Chinese middle-school students.

## 2 Methods

### 2.1 Literature search strategy

Efforts were made to identify all relevant articles published between inception to November 2017. The databases searched were PubMed, Embase, Web of Science, CBM (Chinese database), Chinese National Knowledge Infrastructure (CNKI), Wanfang Data, and the Weipu database. The search terms included: *self-injury/self-harm/self-abuse/nonsuicidal self-injury/deliberate self-harm, adolescen∗/youth/teen/students,* and *China/Chinese*. An example of a full database search is shown below.

#1 (self-injury OR self-harm OR self-abuse OR nonsuicidal self-injury ORdeliberate self-harm).ab,ti.
#2(adolescen∗ OR youth OR teen OR students).ab,ti.
#3 China OR Chinese
#4 #1 AND #2 AND #3

### 2.2 Inclusion and exclusion criteria

Studies were included if they were: published between January 2000 and December 2017; consisted of a cross-sectional study or prevalence study aimed at Chinese middle-school students; contained data on NSSI incidence or provided sufficient information to calculate effect sizes; and were published in the Chinese or English languages. Any studies that did not meet the inclusion criteria were excluded.

### 2.3 Data extraction

Two investigators screened the titles and abstracts of the acquired records, and independently checked the full text for eligibility. Any disagreements were resolved by a third investigator. After removing duplicates, the data were extracted into an electronic spreadsheet. The information recorded included the name of the first author, date of publication, region, sample size, overall prevalence, screening method, screening tools, period prevalence measure (e.g., 6-month NSSI or 12-month NSSI, or lifetime NSSI), number of male and female participants, and number of students reporting NSSI. Where appropriate, we also tried to collect any missing information by communicating with the authors of the original studies.

### 2.4 Quality evaluation

In this study, we used the “Guidelines for critically appraising studies of prevalence or incidence of a health problem” provided by Loney et al^[12]^ to measure the quality of the acquired research. The criteria included the following 8 standards. Are the study design and sampling method appropriate for the research question? Is the sampling frame appropriate? Is the sample size adequate? Are objective, suitable and standard criteria used for measurement of the health outcome? Is the health outcome measured in an unbiased fashion? Is the response rate adequate? Are the refusers described? Are the estimates of prevalence or incidence given with confidence intervals and in detail by subgroup, if appropriate? Are the study subjects and the setting described in detail and similar to those of interest to you? Scores ranged from 0 to 8, where a score of 0 to 4 indicated low quality, 5 to 6 indicated moderate quality, and 7 to 8 indicated high quality.

### 2.5 Statistical analysis

Freeman-Tukey double arcsine method^[13]^ was used to transform prevalence then performed an inverse-variance weighted. The transformed prevalence is weighted very slightly toward 0.5, so studies with prevalence of zero can be included in the analysis. The pooled prevalence is processed as the back-transform of the weighted mean of the transformed proportions. Cochran *Q* and the *I*^2^ statistic were used to detect the heterogeneity between studies. We found *I*^2^ > 50% and *P < *.05, which indicates the presence of substantial heterogeneity. Then random effect model was used to calculate the pooled prevalence and 95% CI. Next, subgroup analysis was used to explore sources of heterogeneity and the prevalence of NSSI with different characters such as sex, grade, study year, sample size, period prevalence measure, and quality score. In addition, meta-regression was used to explore the relationship of these covariates. Each covariate was entered separately in univariate analyses, and then a multivariable meta-regression model was conducted including all covariates. We used funnel plot and Egger test to evaluate publication bias. All the calculations were performed using Stata 14.0.

## 3 Result

### 3.1 Study state

**Figure 1:**
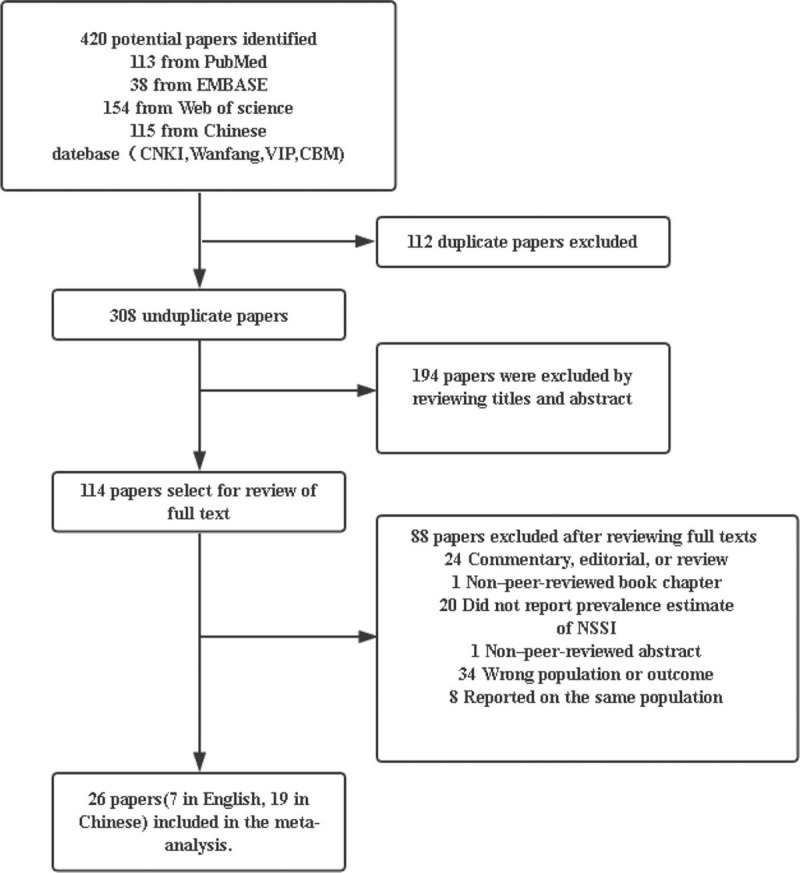
Flow diagram of included/excluded studies.

A total of 420 studies were retrieved. Of these, 26 met the inclusion criteria.^[14–39]^ The screening process is shown in Figure 1. The sample sizes of the 26 papers ranged from 1108 to 25,378, with a total of 160,348 participants. Table 1 shows the characteristics and the quality scores of the 26 studies. All of the selected articles were assessed for methodological quality. Five studies were of high quality and 19 were of moderate quality. Two studies had low quality ratings. The most common problem in the published studies was a lack of explanation of the causes of missing values and invalid questionnaires. Most studies did not report the 95% confidence interval of the prevalence. A few studies did not specify the research object extraction method.

**Table 1:**
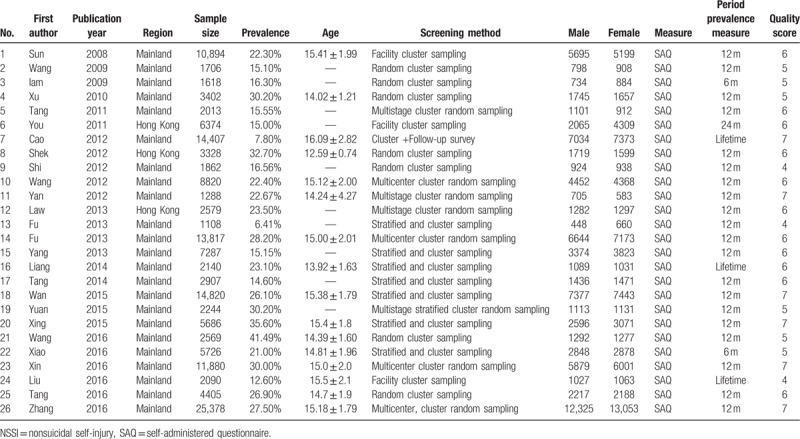
Characteristic of studies on the prevalence of NSSI among Chinese middle school students.

### 3.2 Meta-analysis results

#### 3.2.1 Overall prevalence

**Figure 2:**
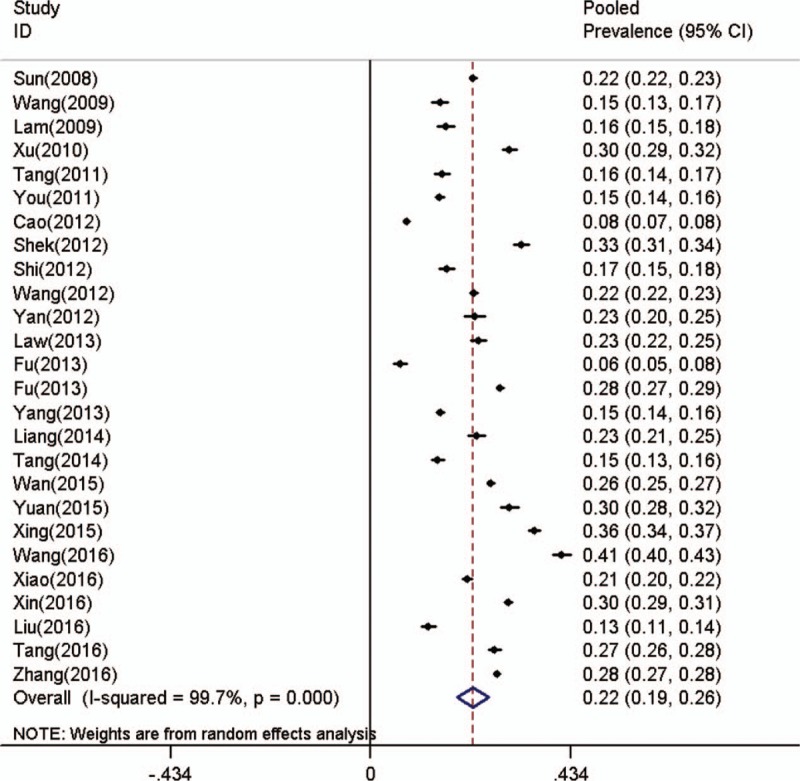
Forest plot of prevalence of NSSI for all participants. NSSI = nonsuicidal self-injury.

Based on the results of the heterogeneity test (*I*^*2*^ = 99.7%, *P < *.0001), a random effects model was used to calculate the pooled prevalence and 95% confidence interval. The overall prevalence of NSSI was 22.37% (95% CI: 18.84%–25.70%, Fig. 2).

### 3.3 Subgroup analysis

The pooled prevalence of NSSI was grouped by study year, sample size, period prevalence measure, female percent, sex, and grade (Table 2). The consolidated prevalence for females (21.9%, 95% CI: 17.6%–26.2%) was higher than that of males (20.6%, 95% CI: 16.1%–25.0%). But the prevalence estimates for studies with more than 50% females (20.1%, 95CI: 15.6%–24.6%) were lower than estimates of groups with less than 50% females (26.4%, 95CI: 22.4%–30.4%). The prevalence in the junior high school group (22.5%, 95% CI: 17.0%–28.0%) was smaller than that of the senior high school group (23.0%, 95% CI: 16.9%–29.1%). The prevalence of NSSI behavior also increased with publication year and sample size. Between 2008 and 2011, the pooled prevalence estimate was 18.8% (95%CI: 15.5%–22.1%), which increased to 22.0% (95%CI: 15.2%–28.8%) between 2012 and 2014, and 28.5% (95CI: 25.3%–31.8%) between 2015 and 2017. The pooled prevalence was 15.4% (95CI:10.2%–20.6%) for sample size < 2000, and 23.2% (95CI: 18.0%–28.3%) for sample size >4000. Regarding period prevalence measure, the pooled prevalence was 14.5% (95%CI: 0.06%–22.7%) for lifetime prevalence measure, and 23.3% (95%CI: 20.5%–26.1%) for 6 to 24 months prevalence measure.

**Table 2:**
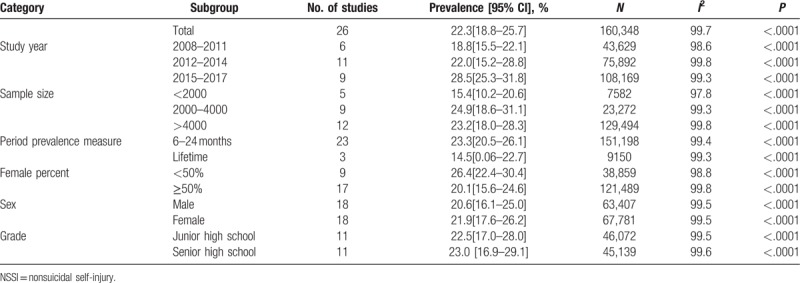
Prevalence of NSSI behavior among middle school students according to different categories.

### 3.4 Meta-regression analysis

We found significant heterogeneity within subgroups (*I*^2^ = 97.8%–99.8%, *P < *.001). Thus, a univariate meta-regression analysis was conducted using the year of publication, female proportion, sample size, period prevalence measure, and quality score as covariates. In 3 publication year groups, the prevalence estimates in year between 2015 and 2017 was significantly higher compared with the other 2 groups (*P = *.011). The pooled prevalence for sample size < 2000 was significantly lower than the other 2 groups (*P = *.043). Multivariate meta-regression was conducted including publication year (2015–2017), female percent, sample size (<2000) and period prevalence measure (6–24 months). This model explained some of the heterogeneity between studies (*R*^2^ = 36.16%, *P = *.039, Table 3).

**Table 3:**
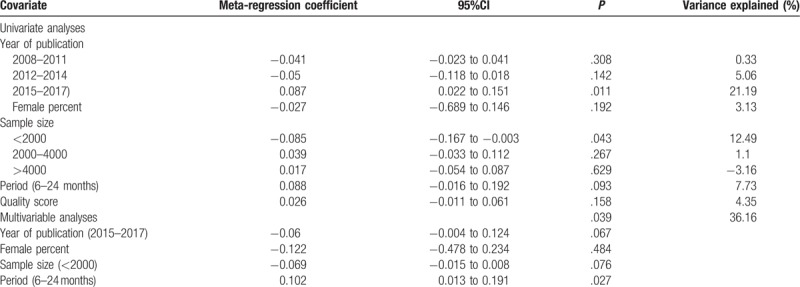
Results of meta-regression for prevalence among Chinese middle-school students.

### 3.5 Sensitivity analysis

There was no significant change in prevalence when using the fix effect model. After removing 2 studies with the lowest scores on the quality evaluation (4 points), the pooled prevalence of NSSI changed from 22.37% (95% CI: 18.84%–25.70%) to 23.33% (95% CI: 19.86%–26.90%).

### 3.6 Publication bias assessment

**Figure 3:**
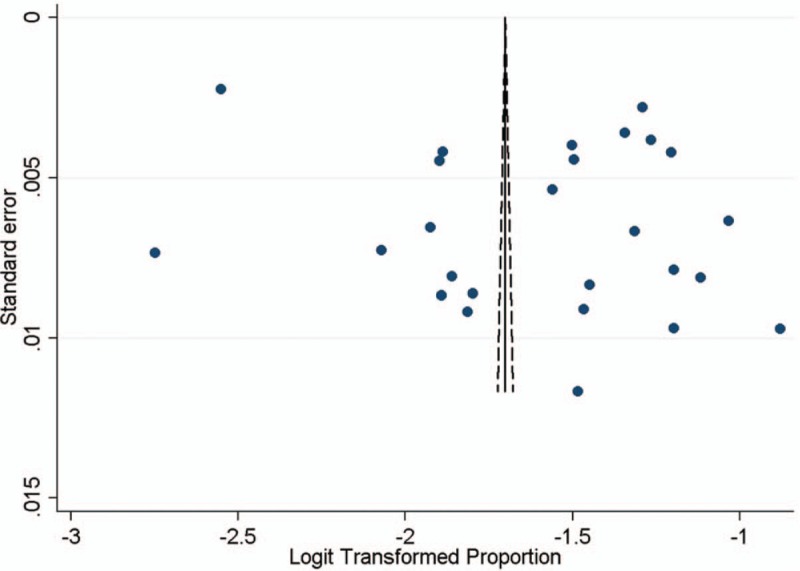
Funnel plot of studies included in meta-analysis.

Funnel plot and Egger's test were used to evaluate publication bias. Although the funnel plot showed marked asymmetry (Fig. 3), but Egger's test indicated no significant publication bias (*P = *.131).

## 4 Discussion

This meta-analysis involving 26 studies and more than 150,000 middle-school students; in this way, we were able to obtain a credible estimate of prevalence. The acquired prevalence of NSSI behaviors in middle-school students, according to our analysis, is 22.37% (95% CI: 18.84%–25.70%). Our results suggested that 1 in 5 middle-school students had NSSI behavior in China.

The prevalence of NSSI among adolescents varied in different regions. For example, in Australia, Tatnell et al^[40]^ found that the prevalence of NSSI was 9.4% among 12 to 15-years old adolescents. Young et al^[41]^ surveyed young people aged 11 to 29 years in Scotland and found that the prevalence of lifetime NSSI among those was 7.1%. These results were lower than our result in this meta-analysis. However, Brunner et al^[42]^ who conducted a cross-sectional study among 12,068 adolescents in 11 European countries, found that the prevalence of NSSI was 27.6%. In Germany, Plener et al^[43]^ assessed the prevalence of NSSI among 665 students in grades 9, and found the prevalence was 25.6%. These studies were basically matched with our result.

Sex may significantly affect the prevalence of NSSI. The results of a meta-analysis showed that women were significantly more likely to report a history of NSSI than men.^[10]^ However, the difference in NSSI prevalence between male and female students did not reach statistical significance in our study, which is consistent with some studies.^[44,45]^ This discrepancy can be partly explained by the following factors. First, 8 studies did not report data by gender, which may have led to bias. Furthermore, due to diversities in methods applied across gender, some studies may have been biased toward higher prevalence of females. Females reported cutting themselves more than males, which is the stereotypical NSSI behavior, while males usually reported self-battery as NSSI behavior.^[45]^ Many early researches restricted their investigations to cutting, which may have ignored a large proportion of NSSI among males.

With respect to publication year, we found that prevalence increased over time. All 26 studies were published between 2008 and 2016. During this period, society was progressing, technology was developing, and pressure was also increasing.^[46]^ We also found that the prevalence increased with sample size, indicating the importance of an appropriate sample size.

Meta-regression revealed that 6 to 24 months prevalence of NSSI was higher than lifetime prevalence, which contrary to our expectations. This unexpected result may be related to recall bias. Furthermore, there were only 3 studies reported the lifetime prevalence of NSSI, which may have led to inaccuracy of the pooled prevalence.

There are several limitations to this study. First, although the sampling method used strict criteria to limit the inclusion of studies, heterogeneity still existed among the included studies. Second, some variables related to NSSI behaviors, such as residence in urban or rural area, and economic conditions, were not reported in most studies, so their mediating effects could not be examined. Third, sensitivity analysis shows that the quality of literature had little effect on the results. In addition, the literature searched was limited to articles published in English or Chinese; thus, it is possible that this meta-analysis did not reflect all outcomes.

## 5 Conclusions

Despite some limitations, this study was a pioneering attempt to estimate the prevalence of NSSI in Chinese middle-school students. The high rate of NSSI deserves immediate management and further attention. The prevalence of NSSI is alarming, considering that it was obtained from a large group of Chinese students. Appropriate mental health education by schools is therefore necessary.

## Author contributions

Junjie Lang contributed to the conception and design of this study and is the guarantor of the study. Yingshui Yao developed the search strategy and provided advice on data analysis and presentation of study results. Both authors approved the final version of the manuscript.

**Data curation:** Junjie Lang.

**Methodology:** Yingshui Yao.
